# Dual action of highbush blueberry proanthocyanidins on *Aggregatibacter actinomycetemcomitans* and the host inflammatory response

**DOI:** 10.1186/s12906-017-2072-x

**Published:** 2018-01-10

**Authors:** Amel Ben Lagha, Geneviève LeBel, Daniel Grenier

**Affiliations:** Oral Ecology Research Group (GREB), Faculty of Dentistry, Université Laval, 2420 Rue de la Terrasse, Quebec City, QC G1V 0A6 Canada

**Keywords:** *Aggregatibacter actinomycetemcomitans*, Blueberry, Cytokine, Keratinocyte, Leukotoxin, Macrophages, Matrix metalloproteinase, Periodontal disease, Proanthocyanidins, Tight junction

## Abstract

**Background:**

The highbush blueberry (*Vaccinium corymbosum*) has a beneficial effect on several aspects of human health. The present study investigated the effects of highbush blueberry proanthocyanidins (PACs) on the virulence properties of *Aggregatibacter actinomycetemcomitans* and macrophage-associated inflammatory responses.

**Methods:**

PACs were isolated from frozen highbush blueberries using solid-phase chromatography. A microplate dilution assay was performed to determine the effect of highbush blueberry PACs on *A. actinomycetemcomitans* growth as well as biofilm formation stained with crystal violet. Tight junction integrity of oral keratinocytes was assessed by measuring the transepithelial electrical resistance (TER), while macrophage viability was determined with a colorimetric MTT assay. Pro-inflammatory cytokine and MMP secretion by *A. actinomycetemcomitans*-stimulated macrophages was quantified by ELISA. The U937-3xκB-LUC monocyte cell line transfected with a luciferase reporter gene was used to monitor NF-κB activation.

**Results:**

Highbush blueberry PACs reduced the growth of *A. actinomycetemcomitans* and prevented biofilm formation at sub-inhibitory concentrations. The treatment of pre-formed biofilms with the PACs resulted in a loss of bacterial viability. The antibacterial activity of the PACs appeared to involve damage to the bacterial cell membrane. The PACs protected the oral keratinocytes barrier integrity from damage caused by *A. actinomycetemcomitans*. The PACs also protected macrophages from the deleterious effect of leukotoxin Ltx-A and dose-dependently inhibited the secretion of pro-inflammatory cytokines (IL-1β, IL-6, CXCL8, TNF-α), matrix metalloproteinases (MMP-3, MMP-9), and sTREM-1 by *A. actinomycetemcomitans*-treated macrophages. The PACs also inhibited the activation of the NF-κB signaling pathway.

**Conclusion:**

The antibacterial and anti-inflammatory properties of highbush blueberry PACs as well as their ability to protect the oral keratinocyte barrier and neutralize leukotoxin activity suggest that they may be promising candidates as novel therapeutic agents.

## Background

Periodontal disease is a biofilm-induced chronic inflammatory disease characterized by the loss of the tooth-supporting tissues, including alveolar bone. The Gram-negative periodontopathogen *Aggregatibacter actinomycetemcomitans* is the key factor in the etiology of localized aggressive periodontitis [1, 2]. This rapidly-progressing form of periodontitis, which affects incisors and first molars in teenagers and young adults, has been linked to a genetic predisposition [3–6]. *A. actinomycetemcomitans* has also been associated with non-oral infections such as endocarditis, septicemia, pneumonia, infectious arthritis, osteomyelitis, and various types of abscesses [3–6].

*A. actinomycetemcomitans* expresses several virulence factors that enable it to colonize and invade oral tissues, avoid the host defense system, and cause tissue destruction [5]. Its ability to adhere to biotic and abiotic surfaces is an important factor for host colonization [7, 8]. Strains or mutants that are unable to adhere to oral tissues are also unable to colonize the oral cavity of the rat and to cause bone loss in a rat model of periodontitis [9, 10]. *A. actinomycetemcomitans* can form biofilms that protect it from antibiotics commonly used in periodontal therapy [11]. It secretes a leukotoxin (LtxA) that selectively kills human leukocytes by inducing apoptosis and lysis [12]. It has also been suggested that there is an association between the leukotoxicity of *A. actinomycetemcomitans* and aggressive forms of periodontitis [12].

The gingival epithelium comprises the epithelial tissue that covers the external surface of the gingiva, the epithelium lining the gingival sulcus, and the junctional epithelium [13]. This structure plays a crucial protective role, acting as a mechanical and antimicrobial barrier that prevents the invasion of the periodontium by periodontopathogens [13]. Inflammation of the periodontium begins with the penetration of bacteria or their products through the epithelial lining [14, 15]. Macrophages and monocytes are the first line of host defenses against periodontal infections and play a key role in the initiation of an adaptive immune response [16]. When these cells are stimulated with *A. actinomycetemcomitans*, they secrete many pro-inflammatory cytokines, including tumor necrosis factor α (TNF-α), interleukin-1β (IL-1β), interleukin-6 (IL-6), and interleukin-8 (CXCL8) [17]. While the host response is involved in gingival tissue homeostasis, uncontrolled and excessive stimulation may lead to a chronic inflammatory state due to the secretion of large amounts of inflammatory mediators and matrix metalloproteinases (MMPs) by mucosal and immune cells, contributing to periodontal tissue destruction [18, 19].

The beneficial effects of bioactive plant food compounds on periodontal diseases have received considerable attention over the past 10 years [20]. Based on our current understanding of the etiology and pathogenesis of periodontitis, plant polyphenols may be highly promising candidates for use in adjunctive periodontal therapies. The highbush blueberry (*Vaccinium corymbosum* L.) is cultivated in many regions of the world and is one of the most commonly consumed berries in the United States, ranking second after strawberries in popularity [21]. Many studies have shown that blueberries have a positive effect on brain aging, diabetes, various cancers, and cardiovascular diseases [22–24]. Blueberries contain significant amounts of flavonoids, including anthocyanins, flavanols, flavonols, and proanthocyanidins (PACs) [25, 26], which likely contribute to the reported beneficial health effects in humans. PACs are some of the most abundant compounds in tea, apples, and berries. They are oligomers of catechin and epicatechin and their gallic acid esters. In the present study, we investigated the antibacterial and anti-biofilm effects of highbush blueberry PACs (hereafter simply referred to as PACs) against *A. actinomycetemcomitans* as well as the protective effects of PACs with regard to the breakdown of the integrity of the epithelial tight junction by *A. actinomycetemcomitans* as well as macrophage killing by the leukotoxin produced by this periodontopathogen. We also assessed the anti-inflammatory effects of PACs by evaluating the inhibition of NF-κB activation and cytokine/MMP secretion by monocytes/macrophages.

## Methods

### Preparation of highbush blueberry PACs

PACs were isolated from frozen highbush blueberries (*V. corymbosum* L.), produced by the Marucci Center for Blueberry and Cranberry Research (Rutgers University, Chatsworth, NJ), using solid-phase chromatography according to a well-established method for PAC isolation [27]. Electrospray mass spectrometry, ^13^C NMR, matrix-assisted laser desorption/ionization time-of-flight mass spectrometry and acid catalyzed degradation with phloroglucinol confirmed the purity of the PAC fraction [27]. Purified PACs were dissolved in 50% ethanol at a final concentration of 10 mg/ml and were stored at 4 °C in the dark for up to 1 month. Preliminary experiments showed that at the dilutions used, the ethanol added had no effects on the assay described below.

### Bacteria and lipopolysaccharide preparation

*A. actinomycetemcomitans* ATCC 29522 was grown anaerobically (80% N_2_:10% CO_2_:10% H_2_) for 24 h at 37 °C in Todd-Hewitt broth (THB; BD Diagnostics, USA) supplemented with 1% yeast extract. *A. actinomycetemcomitans* LPS was isolated using the protocol described by Darveau and Hancock [28]. It was dissolved at a concentration of 1 mg/ml in sterile distilled water and was stored at −20 °C until used.

### Biofilm formation

#### Microplate assay

*A. actinomycetemcomitans* was grown in the wells of a 96-well microplate for 24 h under anaerobic conditions in the absence or presence of two-fold serial dilutions of PACs (500 to 3.9 μg/ml). Bacterial growth was monitored by recording the optical density at 660 nm (OD_660_) using a Synergy 2 microplate reader (BioTek Instruments, USA). Thereafter, medium containing free-floating bacteria was removed by aspiration using a 26G needle. The wells were washed three times with distilled water, and the biofilms were stained for 15 min with 100 μl of 0.05% crystal violet. The wells were then rinsed three times with distilled water and were dried for 2 h at 37 °C. Ethanol (100 μl, 95% [*v*/v]) was added to each well, and the plate was shaken for 10 min to release the dye from the biofilms. The absorbance at 550 nm (A_550_) was recorded using a Synergy 2 microplate reader.

#### Scanning electron microscopy

The effect of PACs on *A. actinomycetemcomitans* biofilm formation was also examined by scanning electron microscopy. An overnight culture of *A. actinomycetemcomitans* suspended in fresh culture medium to an OD_660_ of 0.2 in the absence or presence of 31.25 or 62.5 μg/ml of PACs was added (1 ml) to the wells of a 12-well microplate containing a sterile 13-mm-diameter plastic coverslip. After a 24-h incubation under anaerobiosis, the medium and free-floating bacteria were removed by aspiration, and the plastic coverslips were washed twice with 0.1 M cacodylate buffer (pH 7.2). The biofilm-coated coverslips were incubated for 3 h in fixation buffer (2.5% [*w*/*v*] glutaraldehyde [grade I] and 1 mM CaCl_2_ in 0.1 M cacodylate buffer, pH 7.2), washed three times (20 min each time) with 0.1 M cacodylate buffer (pH 7.2), and post-fixed for 90 min at room temperature in 1% [*w*/*v*] osmic acid containing 2 mM potassium ferrocyanide and 6% [w/v] sucrose in cacodylate buffer. The samples were dehydrated using a graded series of ethanol (50, 70, 95, 100%), critical point-dried, gold-sputtered, and examined using a JEOL JSM6360LV scanning electron microscope operating at 30 kV.

### Biofilm viability and eradication

We investigated the capacity of PACs to eradicate biofilms and kill biofilm-embedded bacteria. Briefly, 24-h *A. actinomycetemcomitans* pre-formed biofilms in the wells of a 96-well microplate were washed once with 50 mM phosphate-buffered saline pH 7.2 (PBS) and were treated for 6 h under anaerobic conditions with PACs (500 to 31.25 μg/ml). In order to assess biofilm eradication, a series of treated biofilms was washed twice with PBS and was stained with crystal violet as described above. A second series of treated biofilms was used to assess bacterial viability using a BacTiter-Glo™ microbial cell viability assay kit (Promega Corporation, USA) in accordance with the manufacturer’s protocol. Luminescence was monitored using a Synergy 2 microplate reader. Biofilms treated with PBS were used as controls.

### Cell membrane permeability assay

The effect of PACs on the integrity of the *A. actinomycetemcomitans* membrane was determined using the intracellular dye calcein acetoxymethyl ester (calcein-AM) (Sigma-Aldrich Canada Co., Canada), as previously described [29]. Briefly, *A. actinomycetemcomitans* cells from a 10-ml overnight culture were suspended in 5 ml of PBS and were incubated anaerobically in the presence of 25 μl of 1 mM calcein-AM for 2 h at room temperature. The bacterial cells were then washed twice and were suspended to an OD_660_ of 0.2 in PBS. Calcein-AM-loaded bacteria were placed (100 μl) in the wells of a black wall black bottom 96-well microplate (Greiner Bio-One North America Inc., USA) and were incubated at room temperature in the presence of two-fold serial dilutions of PACs (250 to 31.25 μg/ml). The release of calcein-AM caused by cell damage was monitored every 10 min for 240 min using a microplate reader, with the excitation wavelength set at 485 nm and the emission wavelength set at 530 nm. PBS was used as a negative control while cells treated with chlorhexidine (200 μg/ml) were used as a positive control.

### Oral keratinocyte tight junction integrity

The previously characterized oral keratinocyte cell line B11 [30], which was kindly provided by S. Groeger (Justus-Liebig-University Giessen, Germany), was used to evaluate the effect of PACs on the integrity of the oral keratinocyte tight junction by determining the transepithelial electrical resistance (TER) [31]. Keratinocytes were cultured in keratinocyte serum-free medium (K-SFM; Life Technologies Inc., Canada) supplemented with growth factors (50 μg/ml of bovine pituitary extract and 5 ng/ml of human epidermal growth factor) and 100 μg/ml of penicillin G-streptomycin at 37 °C in a 5% CO_2_ atmosphere. Keratinocytes (3 × 10^5^ cells per insert) were seeded on Costar™ Transwell™ clear polyester membrane inserts (6.5 mm in diameter, 0.4-μm pore size; Corning Co., USA). The basolateral and apical compartments were filled with 0.6 ml and 0.1 ml of culture medium, respectively. Following a 72-h incubation, the conditioned medium was replaced with antibiotic-free K-SFM and the keratinocytes were incubated for a further 16 h. The TER values were then measured using an ohm/voltmeter (EVOM2; World Precision Instruments, USA) after 0, 24, 48, and 72 h. Resistance values were calculated in Ohms (Ω)/cm^2^ by multiplying the resistance values by the surface area of the membrane filter. Results are expressed as the percentage of the basal control value measured at time 0 h (100% value). *A. actinomycetemcomitans* cells at multiplicity of infections (MOIs) of 10^3^ and 10^4^ were added to the medium in the apical compartment to determine the effect of *A. actinomycetemcomitans* on the integrity of the oral keratinocyte tight junction. The effect of combining PACs (125 to 31.25 μg/ml) and *A. actinomycetemcomitans* (MOI of 10^4^) was also tested. These concentrations were used based on preliminary assays showing that ≤125 μg/ml of PACs had no effect on the viability of B11 cells as determined using an MTT (3-[4,5-diethylthiazol-2-yl]-2,5diphenyltetrazolium bromide) colorimetric assay (Roche Diagnostics, Canada).

### Immunofluorescent staining of zonula occludens-1 and occludin

The oral keratinocytes treated for 24 h as described above were immunostained for two tight junction proteins, zonula occludens-1 and occludin. Briefly, cells were fixed in PBS containing 4% paraformaldhehyde for 20 min, permeabilized with 0.1% Triton-X100 for 10 min, and blocked in 3% nonfat milk in 20 mM Tris hydrochloride buffer - 150 mM NaCl - 0.5% Tween 20 (pH 8) for 40 min. The cells were labeled with anti-occludin antibody - Alexa Fluor® 488 (331511) conjugate and anti-ZO-1 antibody - Alexa Fluor® 594 conjugate (ZO-1-1A12) at 2.5 μg/ml in blocking buffer for overnight at 4 °C. Then, the cells were washed with PBS and treated with the ProLong® Diamond antifade (Life Technologies Inc.,), and the slides were sealed with a coverslip using nail polish and kept in the dark at 4 °C. After washing with PBS, the localization of tight junction proteins in B11 cells was visualized using an Olympus FSX100 fluorescence microscope and FSX-BSW imaging software (Olympus, Tokyo, Japan).

### Purification of LtxA from *A. actinomycetemcomitans*

Leukotoxin (LtxA) was purified from a culture supernatant of *A. actinomycetemcomitans* strain JP2 as previously described [32]. The protein concentration of the purified LtxA was determined using a Pierce™ BCA Protein assay kit according to the manufacturer’s protocol (Thermo Fisher Scientific, Canada). The purity of the LtxA preparation was assessed by SDS-12% polyacrylamide gel electrophoresis (PAGE) and Coomassie blue staining.

### Effect of LtxA on macrophage viability

U937 human monocytes (American Type Culture Collection, USA; CRL-1593.2) were cultivated in Roswell Park Memorial Institute 1640 medium (RPMI-1640; Life Technologies Inc.) supplemented with 10% heat-inactivated fetal bovine serum (FBS) and 100 μg/ml of penicillin G/streptomycin at 37 °C in a 5% CO_2_ atmosphere. The monocytes (2.5 × 10^5^ cells/ml) were incubated in RPMI-10% FBS containing 100 ng/ml of phorbol myristic acid (PMA; Sigma-Aldrich, Canada) for 48 h to induce differentiation into adherent macrophage-like cells [33]. The adherent macrophage-like cells were detached by scraping, and were washed, suspended in RPMI-1% FBS at a concentration of 1 × 10^6^ cells/ml, seeded into the wells of a 96-well microplate (1 × 10^5^ cells/well), and incubated overnight at 37 °C in a 5% CO_2_ atmosphere. To evaluate the ability of PACs to prevent LtxA-induced cytolysis, the macrophage-like cells were incubated for 60 min at 37 °C in a 5% CO_2_ atmosphere in the presence of 1 μg/ml of LtxA together with serial dilutions of PACs (500 to 3.9 μg/ml; in RPMI supplemented with 1% FBS). Wells with no LtxA or PACs were used as controls. LtxA-induced cell death was monitored using the colorimetric MTT assay.

### Cytokine and MMP secretion by macrophages

Adherent macrophage-like cells were prepared and were harvested as described above. The cells were suspended in RPMI-1% FBS at a concentration of 1 × 10^6^ cells/ml, seeded in the wells of a 12-well microplate (1 × 10^6^ cells/well), and incubated overnight at 37 °C in a 5% CO_2_ atmosphere. To exclude the possibility that PAC-associated toxicity might cause a decrease in cytokine and MMP levels, the viability of PAC-treated macrophages was assessed using an MTT assay. The macrophage-like cells were pre-treated for 2 h with PACs (125 to 31.25 μg/ml) prior to being stimulated with *A. actinomycetemcomitans* at an MOI of 100. An assay using a commercial inhibitor (BAY-11-7082; 25 μM; EMD Millipore Canada, Canada) was used as a positive control for the inhibition of cytokine and MMP secretion. Cells incubated in culture medium with or without PACs and stimulated or not with bacteria were used as controls. After a 24-h incubation at 37 °C in a 5% CO_2_ atmosphere, the culture medium supernatants were collected and were stored at −20 °C until used. Enzyme-linked immunosorbent assay (ELISA) kits (eBioscience Inc., USA, and R&D Systems, USA) were used to determine IL-1β, IL-6, CXCL8, TNF-α, MMP-3, and MMP-9 concentrations according to the manufacturers’ protocols.

### Activation of the NF-κB transcription factor

The human monoblastic leukemia cell line U937 3xκB-LUC, a subclone of the U937 cell line stably transfected with a luciferase gene coupled to a promoter of three NF-κB-binding sites, was kindly provided by R. Blomhoff (University of Oslo, Norway) [34]. The cells were routinely cultivated in RPMI-1640 supplemented with 10% heat-inactivated FBS, 100 μg/ml of penicillin G/streptomycin, and 75 μg/ml of hygromycin B at 37 °C in a 5% CO_2_ atmosphere. The effect of two-fold serial dilutions (500 to 3.9 μg/ml) of PACs on U937 3xκB-LUC viability was first determined using an MTT assay to identify non-cytotoxic concentrations. To induce the activation of the NF-κB transcription factor, the U937 3xκB-LUC cells (10^6^ cells/well) were seeded in the wells of a black wall, black bottom 96-well microplate and were stimulated for 6 h with *A. actinomycetemcomitans* LPS (1, 10, 100, and 1000 ng/ml). To investigate the effect of PACs on *A. actinomycetemcomitans* LPS-induced NF-κB activation, U937 3xκB-LUC cells were pre-incubated with two-fold serial dilutions of PACs (125 to 3.9 μg/ml; in RPMI-1% FBS) for 30 min and were then stimulated for 6 h with LPS (1 μg/ml). Wells with no LPS or PACs were used as controls to determine basal NF-κB activity. An assay using the commercial inhibitor BAY-11-7082 (25 μM) was used as a positive control for the inhibition of the NF-κB signaling pathway. NF-κB activation was determined by measuring luciferase activity following the addition Bright-Glo™ reagent (Promega Corporation, USA) in accordance with the manufacturer’s protocol. Luminescence was monitored using a Synergy 2 microplate reader.

### Statistical analysis

Unless indicated otherwise, all experiments were performed in triplicate in three independent experiments. The data are expressed as means ± standard deviations (SD). Statistical analyses were performed using a one-way ANOVA analysis of variance with a post hoc Bonferroni multiple comparison test (GraphPad Software Inc., USA). All results were considered statistically significant at *p* < 0.01 or *p* < 0.001.

## Results

**Fig. 1:**
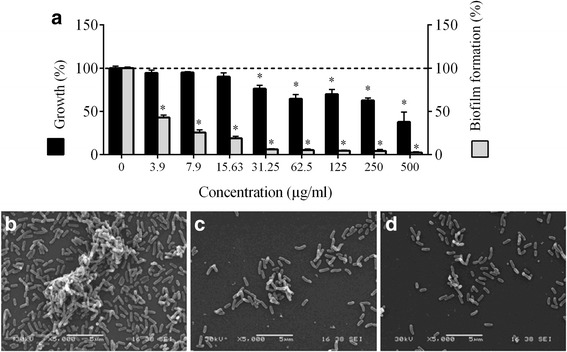
Effect of highbush blueberry PACs on *A. actinomycetemcomitans* growth and biofilm formation (panel **a**). ∗: significantly different (*p* < 0.01) from the control (no PACs). Scanning electron micrographs of *A. actinomycetemcomitans* biofilms formed in the absence (panel **b**) and presence of 31.25 μg/ml (panel **c**) and 62.5 μg/ml of highbush blueberry PACs (panel **d**)

**Fig. 2:**
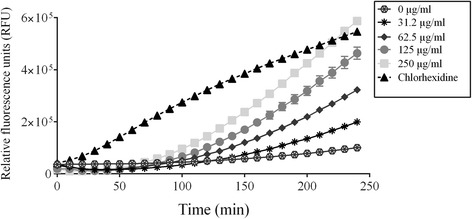
Time-course release of calcein-AM by *A. actinomycetemcomitans* cells treated with various concentrations of highbush blueberry PACs. Chlorhexidine (200 μg/ml) was used as a positive control. A significant (*p* < 0.01) release of calcein-AM was observed with PACs at 62.5, 125, and 250 μg/ml following a treatment of bacteria for 120 min. To obtain the same significance, a treatment of 160 min was necessary when PACs were used at 31.25 μg/ml

The ability of the PACs to interfere with the growth of *A. actinomycetemcomitans* was assessed first. At a concentration of 500 μg/ml, the PACs reduced the growth of *A. actinomycetemcomitans* by 62.5% (Fig. 1a). To identify the mechanism by which PACs exert their antimicrobial activity against *A. actinomycetemcomitans,* we investigated the effect of the PACs on the cytoplasmic membrane integrity of *A. actinomycetemcomitans* using the fluorescent dye calcein-AM. The addition of the PACs to calcein-AM-loaded *A. actinomycetemcomitans* caused a dose- and time-dependent release of fluorescence, suggesting that cell lysis had occurred (Fig. 2). The release of calcein-AM increased 3.73-fold following a 3-h exposure of the bacteria to 125 μg/ml of PACs.

We then tested the effect of the PACs on biofilm formation by *A. actinomycetemcomitans* (Fig. 1a). The PACs at concentrations ranging from 500 to 3.9 μg/ml significantly and dose-dependently reduced biofilm formation. More specifically, 31.25 μg/ml of the PACs reduced the growth of *A. actinomycetemcomitans* by 23.83% and inhibited biofilm formation by 93.98%. The effect of the PACs on biofilm formation was also visualized by scanning electron microscopy. Electron micrographs clearly showed that there is a marked reduction in mature biofilms and that the architecture of the biofilms is disrupted when *A. actinomycetemcomitans* is grown in the presence of the PACs (Fig. 1b).

**Fig. 3:**
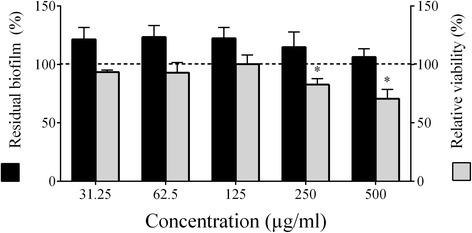
Effect of highbush blueberry PACs on *A. actinomycetemcomitans* biofilm desorption and viability. ∗: significantly different (*p* < 0.01) from the control (no PACs; horizontal line)

Given that the PACs reduced biofilm formation by *A. actinomycetemcomitans*, we then investigated their capacity to promote biofilm desorption and reduce biofilm viability. No desorption was observed when a pre-formed *A. actinomycetemcomitans* biofilm was treated with the PACs (up to 500 μg/ml) for 6 h (Fig. 3). However, the treatment resulted in a dose-dependent loss of biofilm viability. More specifically, 500 μg/ml of the PACs reduced biofilm viability by 29.32%.

**Fig. 4:**
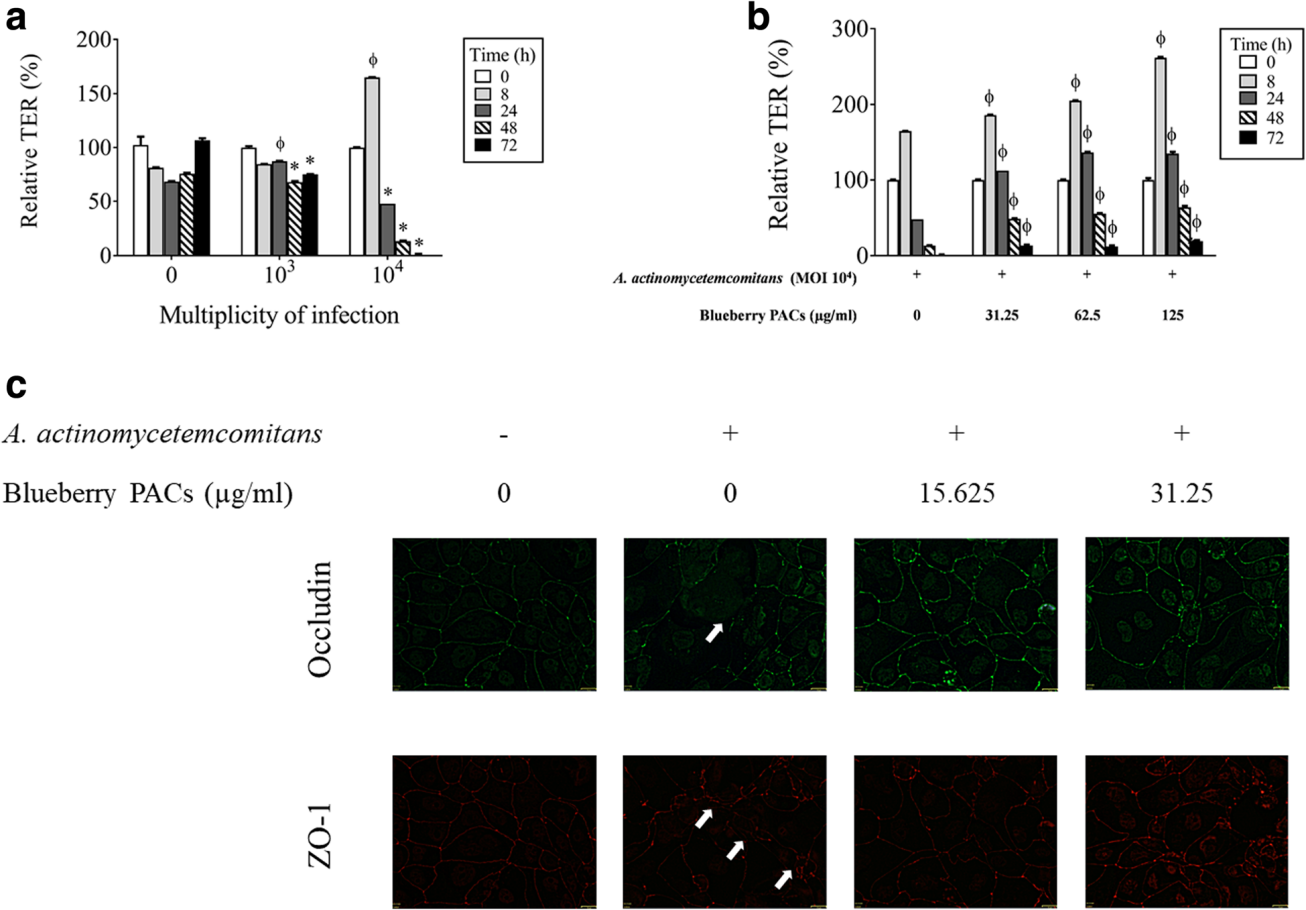
Effect of *A. actinomycetemcomitans* in the absence and presence of highbush blueberry PACs on the integrity of the oral keratinocyte tight junction. Panel **a**: Effects of time and the number of *A. actinomycetemcomitans* cells on the transepithelial electrical resistance (TER) of oral keratinocytes. Panel **b**: Inhibitory effect of highbush blueberry PACs on the *A. actinomycetemcomitans*-induced decrease in the TER of oral keratinocytes. Panel **c**: Immunofluorescence staining of tight junction proteins occludin and zonula occludens-1 (ZO-1) of oral keratinocytes infected (24 h) by *A. actinomycetemcomitans* in the absence and presence of blueberry PACs. A 100% value was assigned to the TER at time 0. Results are expressed as the mean ± SD of triplicate assays. Φ: significant increase (*p* < 0.001) compared with unstimulated control cells (**a**) or with *A. actinomycetemcomitans*-stimulated cells not treated with highbush blueberry PACs (B). ∗: significant decrease (*p* < 0.001) compared with unstimulated control cells

Since periodontopathogens may have deleterious effects on the integrity of the oral keratinocyte tight junction, we determined whether the PACs protect the tight junction from such damage. Figure 4a shows the effect of *A. actinomycetemcomitans* at MOIs of 10^3^ and 10^4^ on the TER of keratinocytes following apical stimulation with bacteria. The TER values decreased in a concentration- and time-dependent manner in the presence of *A. actinomycetemcomitans.* Following a 48-h incubation, *A. actinomycetemcomitans* at an MOI of 10^4^ decreased the TER by 82.43% while an MOI of 10^3^ decreased the TER by 10.28%. We then examined the protective effect of the PACs when keratinocytes are challenged with *A. actinomycetemcomitans* at an MOI of 10^4^. As shown in Fig. 4b, the PACs attenuated the effect of *A. actinomycetemcomitans* in a concentration- and time-dependent manner. For example, 62.5 μg/ml of the PACs reduced the ability of *A. actinomycetemcomitans* to decrease the TER by 2.9-fold and 4.1-fold after 24 and 48 h, respectively.

Immunostaining of ZO-1 and occludin was performed to determine whether *A. actinomycetemcomitans* affect the oral keratinocyte barrier integrity through disruption of these tight junction proteins. As shown in Fig. 4c, a 24-h treatment of keratinocytes with *A. actinomycetemcomitans* (MOI of 10^4^) was associated with a decreased labeling of both ZO-1 and occludin However, the presence of PACs prevented this decreased labeling. At a concentration of 31.25 μg/ml, PACs allowed a marked labeling suggesting the maintenance of expression of ZO-1 and occludin.

**Fig. 5:**
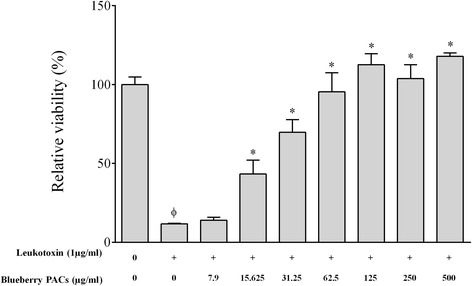
Effect of highbush blueberry PACs on *A. actinomycetemcomitans* leukotoxin (LtxA) activity on macrophage-like cells. Φ: significant loss of cell viability (*p* < 0.001) compared to control cells not treated with LtxA. *: significant increase of cell viability (*p* < 0.001) compared to cells treated with LtxA

*A. actinomycetemcomitans* LtxA is considered a major virulence factor because of its ability to kill macrophages. We thus determined whether the PACs can reduce LtxA cytotoxic activity. We showed that 1 μg/ml of LtxA reduced the viability of macrophage-like cells by 88.26% (Fig. 5), while the PACs exhibited a marked capacity to attenuate LtxA activity. More specifically, 125, 62.5, and 31.25 μg/ml of the PACs reduced the toxicity of LtxA by 100%, 95.4%, and 69.70%, respectively (Fig. 5).

**Fig. 6:**
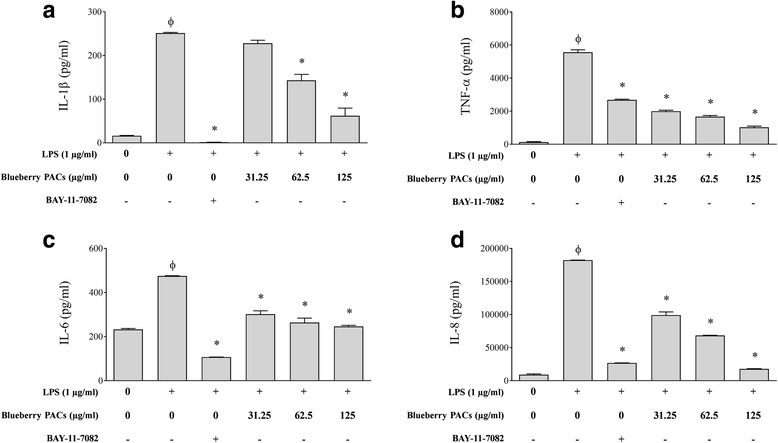
Effect of highbush blueberry PACs on the secretion of **a** IL-1β, **b** TNF-α, **c** IL-6, and **d** CXCL8 by macrophages stimulated with *A. actinomycetemcomitans* LPS*.* The commercial inhibitor BAY-11-7082 (25 μM) was used as a positive inhibitory control. Results are expressed as the means ± SD of triplicate assays from three independent experiments. Φ: significant increase (*p* < 0.001) compared to unstimulated control cells. *: significant decrease (*p* < 0.001) compared to LPS-stimulated cells not treated with highbush blueberry PACs

**Fig. 7:**
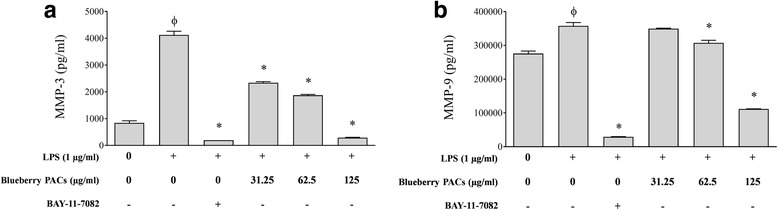
Effect of highbush blueberry PACs on the secretion of **a** MMP-3 and **b** MMP-9 by macrophages stimulated with *A. actinomycetemcomitans* LPS*.* The commercial inhibitor BAY-11-7082 (25 μM) was used as a positive inhibitory control. Results are expressed as the means ± SD of triplicate assays from three independent experiments. Φ: significant increase (*p* < 0.001) compared to unstimulated control cells. *: significant decrease (*p* < 0.001) compared to LPS-stimulated cells not treated with highbush blueberry PACs

We investigated the ability of the PACs to reduce the *A. actinomycetemcomitans* LPS-induced inflammatory response of macrophages. Adherent macrophage-like cells were pre-treated for 2 h with the PACs and were then stimulated for 24 h with 1 μg/ml of LPS. Secreted pro-inflammatory cytokines (IL-1β, TNF-α, IL-6 and CXCL8) and MMPs (MMP-3 and MMP-9) were then quantified by ELISA. To exclude the possibility that PAC-related toxicity may have an effect on cytokine and MMP levels, non-cytotoxic concentrations of PACs were determined. PAC concentrations up to 125 μg/ml had no effect on macrophage viability, which was ≥84.40 ± 12.26% compared with the untreated controls (data not shown). The secretion of pro-inflammatory cytokines (IL-1β, TNF-α, IL-6, CXCL8) by macrophages stimulated with *A. actinomycetemcomitans* LPS was significantly and dose-dependently attenuated by the PACs compared to the controls. At a concentration of 125 μg/ml, the PACs reduced the secretion of IL-1β, TNF-α, IL-6, and CXCL8 by 75.34% (Fig. 6a), 81.64% (Fig. 6b), 48.27% (Fig. 6c), and 90.19% (Fig. 6d), respectively. Lastly, MMP-3 and MMP-9 secretion by *A. actinomycetemcomitans* LPS-stimulated macrophages was also attenuated by the PACs, in some cases below basal levels. More specifically, 125 and 62.5 μg/ml of the PACs reduced the secretion of MMP-3 by 93.04% and 54.57%, respectively (Fig. 7a), and the secretion of MMP-9 by 68.78% and 14.07%, respectively (Fig. 7b).

**Fig. 8:**
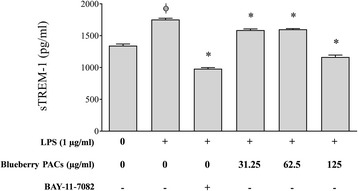
Effect of highbush blueberry PACs on the secretion/shedding of sTREM-1 by macrophages stimulated with *A. actinomycetemcomitans* LPS*.* The commercial inhibitor BAY-11-7082 was used as a positive inhibitory control. Results are expressed as the means ± SD of triplicate assays from three independent experiments. Φ: significant increase (*p* < 0.001) compared to non-stimulated control cells. *: significant decrease (*p* < 0.001) compared to LPS-stimulated cells not treated with highbush blueberry PACs

We also investigated the ability of the PACs to inhibit the secretion/shedding of sTREM-1 induced by the stimulation of macrophages with *A. actinomycetemcomitans* LPS. As shown in Fig. 8, *A. actinomycetemcomitans* LPS significantly increased the secretion/shedding of sTREM-1 (1.3 fold), while 125 and 62.5 μg/ml of the PACs inhibited the secretion/shedding of sTREM-1 by 33.62% and 8.83%, respectively.

**Fig. 9:**
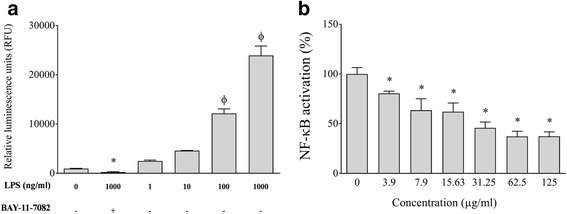
Effect of *A. actinomycetemcomitans* LPS in the absence and presence of highbush blueberry PACs on nuclear factor-κB (NF-κB) activation using the U937-3xκB cell model. Panel **a** Concentration effect of *A. actinomycetemcomitans* LPS on NF-κB activation. Panel **b** Inhibitory effect of highbush blueberry PACs on *A. actinomycetemcomitans* LPS-induced NF-κB activation. A value of 100% was assigned to the activation obtained with *A. actinomycetemcomitans* LPS in the absence of highbush blueberry PACs. BAY-11-7082 was used as a positive inhibitory control. Results are expressed as the mean ± SD of triplicate assays from two independent experiments. Φ: significant increase (*p* < 0.001) compared with nonstimulated control cells. ∗: significant decrease (*p* < 0.001) compared with LPS-stimulated cells not treated with highbush blueberry PACs

Lastly, given that the NF-κB signaling pathway plays a key role in inflammatory processes leading to the secretion of cytokines and MMPs, we investigated the effect of the PACs on the activation of this transcription factor using the U937-3xκB-LUC cell line transfected with a luciferase reporter gene. As shown in Fig. 9a, 1 μg/ml of LPS activated the NF-κB signaling pathway by 24.95-fold. We then determined whether the PACs prevented *A. actinomycetemcomitans* LPS-induced NF-κB activation in U937-3xκB cells. The PACs dose-dependently inhibited the activation of NF-κB signaling pathway induced by LPS. More specially, 125, 62.5, 31.25, and 15.625 μg/ml of the PACs reduced NF-κB activation by 62.69%, 62.82%, 54.26%, and 37.94%, respectively (Fig. 9b). As expected, the commercial inhibitor BAY-11-7082 (25 μM) completely prevented NF-κB activation.

## Discussion

The highbush blueberry (*V. corymbosum* L.), which is mostly grown in the United States, Canada, and South America, has been successfully hybridized with lowbush and rabbiteye blueberry species to create northern and southern varieties, respectively [35]. Many studies have shown that blueberries have beneficial effects on human health, especially with respect to cancers and inflammatory, neurodegenerative, and cardiovascular diseases [36, 37]. The health effects of blueberries have been attributed to their high phenolic acid, catechin (flavanols), and PAC (condensed tannins) content. PACs are oligomers or polymers of polyhydroxy flavan-3-ol units such as (+)-cathechin and (−)-epicathechin [38]. According to Gu et al., highbush blueberries contain 129–230 mg of PACs per 100 g [39]. Previous studies have reported that cranberry PACs interfere with the pathogenic properties of periodontopathogens and have anti-inflammatory properties [40]. Cranberry PACs differ from PACs isolated from other berry fruits, including blueberries, since they are mainly composed of epicatechin subunits with at least one intermolecular A-type bond between O7 and C2 in addition to the carbon-carbon bond [41]. In the present study, we evaluated the ability of PACs isolated from highbush blueberries to attenuate several major virulence properties of *A. actinomycetemcomitans*, a Gram-negative bacterium that is strongly associated with localized aggressive periodontitis [2] as well as with extra-oral infections such as infective endocarditis [42], bacterial arthritis [43], and osteomyelitis [44]. We also investigated the effects of blueberry PACs on the integrity of the oral keratinocyte barrier, leukotoxin activity, and the LPS-mediated inflammatory response of monocytes/macrophages.

Although highbush blueberry PACs (≥ 31.25 μg/ml) significantly reduced the growth of *A. actinomycetemcomitans*, they did not completely inhibit growth, even at the highest concentration tested (500 μg/ml). This finding was consistent with previous studies on the antimicrobial activity of blueberry extracts (highbush, lowbush) against major human pathogens such as *Listeria monocytogenes*, *Helicobacter pylori*, *Salmonella typhinurium*, *Escherichia coli*, and *Fusobacterium nucleatum* [45–49]. Since the bacterial cell membrane is a target for many plant polyphenols, we used a membrane permeabilization assay to show that highbush blueberry PACs caused damage to *A. actinomycetemcomitans* that leads to cell lysis as shown by the release of calcein-AM. A scanning electron microscopic study by Joshi et al. showed that highbush blueberry PACs cause major damage to the cell surface of *Cronobacter sakazakii* [50].

Oral biofilms are structured microbial communities attached to oral surfaces that enable bacteria to evade immune defenses and resist mechanical removal and chemotherapeutic agents [51]. We showed that highbush blueberry PACs possess marked anti-biofilm activity at concentrations that do not inhibit bacterial growth, almost completely preventing *A. actinomycetemcomitans* biofilm formation at concentrations as low as 31.25 μg/ml. The anti-biofilm activity of highbush blueberry PACs was confirmed by scanning electron microscopic observations indicating that they may inhibit the formation and maturation of periodontopathogen biofilms. This result was consistent with our previous study showing that a lowbush blueberry extract inhibited biofilm formation by the periodontopathogen *F. nucleatum* [49]. In the present study, we showed that highbush blueberry PACs decrease the viability of biofilms, without causing biofilm desorption. The mechanism involved in biofilm killing may be related to the ability of these PACs to alter the integrity of the cell membrane, as reported above.

*A. actinomycetemcomitans* colonizes the gingival sulcus by adhering to the sulcular/junctional epithelium. From there, it can invade the epithelium, penetrate into the subgingival connective tissue, and induce inflammatory processes [52]. Based on our understanding of bacteria-mediated periodontal tissue destruction, using agents that maintain the integrity of the oral keratinocyte tight junction may be a valuable strategy for preventing periodontal disease. TER measurements are commonly used to assess the integrity of tight junctions. Using this in vitro model, on the one hand, we observed that incubating the oral keratinocytes with *A. actinomycetemcomitans* (MOIs of 10^4^) results in an increase in TER values within 8 h. On the other hand, the TER values decreased following longer incubation periods (24, 48, and 72 h). Similar observations were made following apical and basolateral *A. actinomycetemcomitans* challenges (data not shown) thus suggesting that the initial increase in TER may not be related to deposition of bacteria on the keratinocyte monolayer. Such increase of TER values was also reported in a previous study of Groeger et al. [52] who showed that stimulation of oral keratinocytes with *Porphyromonas gingivalis* (whole bacteria or supernatant) involved a peak of TER values at 8 h in both primary and immortalized human oral keratinocytes. It has been proposed that this may reflect a defense reaction of host cells, although the underlying mechanisms are still unknown.

We showed that highbush blueberry PACs significantly and dose-dependently reduce the ability of *A. actinomycetemcomitans* to damage the integrity of the keratinocyte tight junction. To the best of our knowledge, this is the first time that *A. actinomycetemcomitans* has been shown to decrease TER values and that highbush blueberry PACs can protect the integrity of the oral keratinocyte barrier. Such a bio-protective effect by polyphenols has been previously reported using an intestinal epithelial barrier model. For example, epigallocatechin-3-gallate (EGCG), quercetin, and resveratrol, which are flavanol-, flavonol-, and stilbene-class polyphenols, respectively, have been reported to protect the barrier function of epithelial intestinal Caco2-cells against indomethacin-induced gastrointestinal damage [53]. Moreover, Park et al. reported that black tea theaflavins increase the integrity of the intestinal barrier by closing the tight junction route in Caco-2 cells [54]. They showed that theaflavins modulate AMP-activated protein kinase, which results in the enhanced expression of tight junction proteins, including occludin, claudin-1, and zonula occludens-1 [54]. In the present study, the immunofluorescence staining brought evidence that the increased keratinocyte barrier integrity in the presence of PACs may result from ZO-1 and occludin over-expression or redistribution. ZO-1 establishes the linkage between transmembrane proteins and the cytoskeleton, whereas occludin is a membrane protein with two extracellular loops that interact with ZO-1. These proteins are known to play a primary role in the maintenance of the epithelial barrier.

*A. actinomycetemcomitans* expresses several virulence factors, including LtxA, an exotoxin that appears to play an important role in the etiopathogenesis of localized aggressive periodontitis [55, 56]. LtxA protects the bacteria from local defense mechanisms by killing leukocytes and disturbing the host response [57, 58]. It is a large pore-forming toxin that belongs to the RTX (Repeats in toxin) family of bacterial proteins [59]. It selectively affects human hematopoietic cells by binding to the lymphocyte function-associated receptor 1 (LFA-1), which disrupts the integrity of the cell membrane [60]. Human monocytes/macrophages are highly sensitive to LtxA. Kelk et al. reported that the LtxA-induced cell death of macrophages occurs through a process leading to a specific and excessive pro-inflammatory response involving the secretion of IL-1β and IL-18 [61]. Since leukotoxicity is correlated with attachment loss in teenagers affected with localized aggressive periodontitis [62], LtxA may be a promising target for future therapeutics. We showed that high concentrations of highbush blueberry PACs completely protect macrophage-like cells. To the best of our knowledge, this is the first report on the potential of polyphenols to neutralize the leukotoxin activity of *A. actinomycetemcomitans*.

Periodontal health requires a balanced immuno-inflammatory state that maintains a host-bacteria homeostasis in the periodontium [63]. Pro-inflammatory cytokines with potent pro-resorptive effects such as IL-1β, TNF-α, and IL-6 are strongly upregulated by *A. actinomycetemcomitans* and likely promote osteoclast formation and bone resorption [64–68]*. A. actinomycetemcomitans* LPS is a key virulence factor that upregulates the pathophysiologic inflammatory response [69]. Such an immune response perturbs normal periodontal tissue remodeling/turnover and ultimately has deleterious effects on periodontal tissue homeostasis [70]. The LPS/TLR4 signaling pathway induces the activation of several innate immune pathways such as phagocytosis and the overexpression of pro-inflammatory chemokines, cytokines and co-stimulatory molecules, which leads to the initiation of the inflammatory response [71–73]. We used macrophage-like cells to show that highbush blueberry PACs significantly reduce the secretion of major pro-inflammatory cytokines (IL-1β, TNF-α, IL-6, and CXCL8) induced by *A. actinomycetemcomitans* LPS. The anti-inflammatory property of PACs is of utmost importance given that periodontitis is caused by inflammophilic pathogens that take advantage of the inflammatory environment in periodontal pockets [74]. The efficient regulation of inflammation is thus likely to control both dysbiosis and disease progression.

The activation of TREM-1 expressed on neutrophils and monocytes amplifies the production of various pro-inflammatory cytokines, chemokines, and cell surface receptors [75]. Moreover, bacterial products such as LPS induce the release of the soluble form of TREM-1 (sTREM-1) in humans [76]. sTREM-1 has also been detected in gingival crevicular fluid and is present in higher concentrations in diseased periodontal sites [77]. However, the exact role of sTREM-1 in the inflammatory cascade remains unclear. Some studies have shown that the upregulation of sTREM-1 production is mediated by bacterial challenges [78] and have concluded that it may be a specific marker of infections in various pathologies [78, 79]. When sTREM-1 is detected in serum it may have been released by circulating leukocytes during the course of a systemic infection [78]. To the best of our knowledge, no studies have investigated the potential of *A. actinomycetemcomitans* LPS to induce the secretion/shedding of sTREM-1. Interestingly, we found that highbush blueberry PACs significantly reduce sTREM-1 levels in a macrophage model stimulated with *A. actinomycetemcomitans* LPS. In a recent study, we reported that tea polyphenols also reduce the secretion of sTREM-1 induced by *F. nucleatum* [49].

Periodontal inflammation associated with persistent chronic bacterial infections in subgingival sites up-regulates the expression and activity of matrix metalloproteinases (MMPs), which contribute to the progressive breakdown of periodontal supporting tissue [80]. MMPs, a large family of calcium-dependent zinc-containing endopeptidases, are involved in tissue remodeling and the degradation of extracellular matrix proteins, including collagens, elastin, matrix glycoprotein, and proteoglycans [81]. While the transcription of MMP genes is rather low in healthy periodontal tissue, the secretion of specific MMPs is up-regulated by various cytokines during periodontitis. For example, IL-1β and TNF-α stimulate the secretion of MMP-3, −8, and −9 by gingival fibroblasts [82]. Moreover, *A. actinomycetemcomitans* has been reported to induce the expression of MMP-1, −2, and −9 in a murine model [83] and in human peripheral blood monocytes [84]. MMPs are considered promising targets for the treatment of periodontal disease due to their involvement in the inflammatory destruction of periodontal attachment. Interestingly, we showed that highbush blueberry PACs reduce MMP-3 and MMP-9 secretion by LPS-stimulated macrophages. MMP-3 (stromelysin) and MMP-9 (gelatinase B) have been strongly associated with the progression of periodontitis [80]. On the one hand, MMP-3 contributes to tissue remodeling and destruction, induces apoptosis, promotes cell differentiation, and activates other latent MMPs such as MMP-8 and MMP-9 [80, 85, 86]. On the other hand, MMP-9 regulates numerous cell activities, such as cell-cell contact, tissue remodeling, cell migration, and cellular differentiation [87].

Lastly, many studies on *A. actinomycetemcomitans* in a rodent model of experimental periodontitis have provided evidence that the NF-κB signaling pathway is involved in the host inflammatory response [70, 88–90]. The activation of NF-κB by nuclear translocation plays a central role in inflammation through its ability to induce the transcription of pro-inflammatory genes [91]. NF-κB activation has been reported to contribute to the pathogenesis of many chronic inflammatory diseases, including rheumatoid arthritis, inflammatory bowel disease, asthma, and oral lichens planus [92–94]. A recent study involving healthy controls and patients with chronic periodontitis showed that the activation of NF-κB (p50/p65) is significantly upregulated in periodontal disease tissues, suggesting that NF-κB inhibitors may be promising agents for managing periodontitis [95]. In this context, we used the U937-3xκB cell model to show that highbush blueberry PACs inhibit NF-κB activation induced by *A. actinomycetemcomitans* LPS; this is likely, at least in part, involved in the reduced secretion of inflammatory mediators by macrophages.

## Conclusions

In conclusion, the antibacterial and anti-inflammatory properties of highbush blueberry PACs and their ability protect the integrity of the oral keratinocyte barrier and to neutralize leukotoxin activity suggest that they may be promising candidates for novel therapeutic agents. The beneficial properties of blueberry PACs identified in this study should open the door for future clinical trials on the potential of these bioactive PACs for periodontal disease prevention and/or treatment. These studies should evaluate the impact of blueberry consumption (fruit, juice, extract) on the severity and progression of periodontal disease. Given that highbush blueberries contain 129–230 mg of PACs per 100 g (fresh weight food) [39], it is believed that effective concentrations could be achieved in the oral cavity. Moreover, studies on the benefits provided by the use of oral-hygiene products (mouthrinses and chewing gums) or slow periodontal-release devices (to be inserted in diseased periodontal sites) containing bioactive PACs are also of high interest.
